# 281. Not the sharpest of tools: Procalcitonin and bacterial co-infections in patients admitted with COVID-19

**DOI:** 10.1093/ofid/ofac492.359

**Published:** 2022-12-15

**Authors:** Alfredo J Mena Lora, Fischer Herald, Brenna Lindsey, Stephanie Echeverria, Sorel Lira, Rodrigo M Burgos

**Affiliations:** University of Illinois Chicago, Chicago, Illinois; University of Illinois at Chicago, Chicago, Illinois; University of Illinois at Chicago, Chicago, Illinois; Saint Anthony Hospital, Chicago, Illinois; Saint Anthony Hospital, Chicago, Illinois; University of Illinois at Chicago, Chicago, Illinois

## Abstract

**Background:**

The COVID-19 pandemic has caused record breaking hospitalizations due to respiratory failure. A major challenge in the management of COVID-19 is the difficulty distinguishing COVID-19 from other causes of lower respiratory tract infections (LRTIs) that may require antimicrobial use (AU). Procalcitonin (PCT) has been used to differentiate viral from bacterial causes of LRTIs and clinicians have relied on PCT to use or withhold antimicrobials. However, the utility of PCT in the setting of COVID-19 remains unclear. We seek to define the role of PCT in patients admitted with COVID-19.

**Methods:**

Retrospective cohort study of COVID-19 inpatients with PCT ordered at a 151-bed urban community hospital from March 2020-March 2022. Ranges of PCT were categorized as high ( >5 µg/L), medium (0.25-5 µg/L), and low (< 0.25 µg/L) risk of infection. Co-infection was defined as presence of clinical and microbiological evidence of infection in blood (BSI) or in sputum within 7 days of admission. Late infections were excluded

**Results:**

Of a total 262 cases, 154 (58%) were low-risk, 43 (16%) medium-risk, and 63 (24%) high-risk (Figure 1). AU in the low-risk category was 29% (45), followed by 29% in the moderate and 36% in high-risk categories. 1 BSI caused by Klebsiella pneumoniae in the low-risk category and 1 LRTI caused by Streptococcus pneumoniae in the high-risk category were found, representing 0.6% and 1.5% of samples in those categories. Total documented infection was 0.7% for all cases.

**Figure 1.**
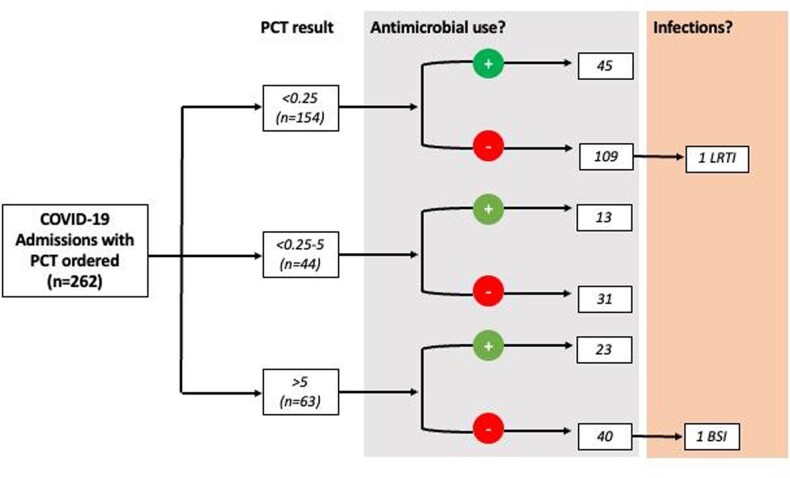
Procalcitonin levels and co-infections in patients admitted with COVID-19 pneumonia.

**Conclusion:**

PCT has limited utility in COVID-19. Co-infection rates on admission are exceedingly rare, representing < 1% of our cohort. Only 2 documented infections were found, 1 of which was in the low-risk category. Thus, PCT was commonly elevated without documented infection. Though rare, a co-infection can occur without elevation of PCT. As described in the 2019 IDSA Community Acquired Pneumonia Guidelines, the use of PCT is of limited utility and may confound providers towards using or deferring antimicrobials inappropriately. This remains true in COVID-19. Antimicrobial stewardship programs should advise against its routine use.

**Disclosures:**

**All Authors**: No reported disclosures.

